# Modeling strategies in non-invasive spinal stimulation: perspectives on state-of-the-art

**DOI:** 10.3389/fnhum.2026.1763470

**Published:** 2026-02-20

**Authors:** Sofia Rita Fernandes

**Affiliations:** Instituto de Biofísica e Engenharia Biomédica, Faculdade de Ciências da Universidade de Lisboa, Campo Grande, Lisbon, Portugal

**Keywords:** direct current, FEM, finite element method, MRI, non-invasive, spinal cord, stimulation, trans-spinal

## Abstract

Non-invasive Spinal Stimulation (NISS) is of increasing interest for clinicians addressing spinal dysfunctions, such as spasticity, chronic pain and hypotonia. NISS can be an alternative when surgically-implanted stimulators or pharmacological therapy are not compatible nor viable. Trans-spinal direct current stimulation (tsDCS) is one NISS strategy that delivers direct currents (DC) of low intensity (1–4 mA) through large electrodes (8–25 cm^2^) placed over selected targets in the vertebral column. Since 2008, tsDCS researchers have build-up evidence regarding modulation of spinal reflexes and sensorimotor responses measured by electromyography. Biophysical constructs based on computational numerical methods provide a well-grounded framework to determine the most effective protocols designed according to each patient’s needs. Additionally, models can work as theoretical labs to investigate how the EF profile induced by stimulation can relate with the changes observed in spinal responses. The accuracy of predictions in tsDCS biophysical constructs strongly rely on how realistic are the digital twins of the spine. The main strategy used is to adapt accurate template models to have a fine description of spinal structures down to the millimeter resolution. However, this strategy lacks the personalized approach of MRI-based realistic models. This is due to the fact that development of pipelines for semi-automatic segmentation of the spinal cord is still in its early stages. This work aims to discuss the current state-of-the art regarding computational constructs of tsDCS, what is known on its effects on spinal networks, based on combined modeling-experimental approaches, and what lies ahead for a more targeted and personalized application.

## 1 Introduction

Non-invasive spinal stimulation (NISS) techniques have drawn increasing attention within the international spinal research community over the last decade, due to its strong potential to modulate spinal cord excitability (Pereira et al., 2022). NISS approaches aim to transiently change spinal circuitry sensorimotor functions in conditions such as spinal cord injury (SCI), chronic pain, and spasticity (Cogiamanian et al., 2012; Hubli et al., 2013). Unlike invasive epidural stimulation, NISS neuromodulatory effects are induced exogenously, offering a safer and more accessible alternative for both clinical and research applications, eliminating the risks commonly associated with surgical procedures and implantation of electrical devices (Hofstoetter and Minassian, 2022; Kuck et al., 2019).

The main technique explored in current state-of-the-art in NISS evolved from non-invasive brain stimulation (NIBS) techniques, specifically transcranial direct current stimulation (tDCS), which already demonstrated the ability to modulate cortical excitability and induce neuroplasticity in the central nervous system with exogenous magnetic and weak electric fields (Nitsche and Paulus, 2000; Lefaucheur et al., 2017). Inspired by these findings, Cogiamanian et al. (2008) used a tDCS device, however placing one of two electrodes over the vertebral column to modulate the spinal cord. When the spinal electrode had anodal polarity, he and his team observed a 25% reduction in the amplitude of the cervico-medullary component of posterior tibial nerve SEPs (P30), demonstrating tsDCS potential to modulate nociceptive responses integrated in the spinal cord. Additionally, the fact that this effect was sustained for 20 min after stimulation, indicates a long lasting effect, possibly of neuroplastic nature, such as observed in tDCS (Nitsche et al., 2008). Other studies followed the same type of procedure, exploring both cervical and thoracolumbar spinal placements, with observed changes in sensorimotor reflex responses (e.g., Winkler et al., 2010; Truini et al., 2011; Lamy et al., 2012; Bocci et al., 2014) and gait recovery (Picelli et al., 2015).

tsDCS induces an electric field (EF) in the spinal tissue that changes polarization of the membrane potential of neurons aligned with the field (Rattay, 1999), resulting in a state change from rest that can facilitate or inhibit neuronal behavior when triggered by a voluntary movement, a sensory integration or a reflex pathway. However, the biophysical mechanisms of these two techniques are very different. tsDCS is essentially conductive, i.e., a weak direct current is applied transcutaneously over the spinal cord using surface electrodes. tsDCS generates low-intensity, constant electric fields (typically less than 1 V/m) that modulate neuronal excitability, with main mechanism identified to be persistent inward currents mediated by calcium channels (Bocci et al., 2015).

The biophysical nature of this technique will influence how the EF will distribute at the target not only in terms of strength, but also in spatial range and spread, tissue and neural elements selectivity, leading to a diversity of effects reported in the literature (Gigliotti and Pereira, 2025). Just as in brain stimulation, computational realistic volume conductor models have been used to simulate and study the effects of NISS and how do these vary with type and placement of electrodes (Fernandes et al., 2018; Fernandes et al., 2019a). Additionally, computational predictions using realistic models of the human trunk and spine inform on how the induced electric field can change with electrical and anatomical characteristics of tissues, enabling optimization of protocol settings for targeted and more effective neuromodulation (Parazzini et al., 2014; Kuck et al., 2017; Kim et al., 2025).

In the field of brain stimulation, there are diverse simulation software available to accurately model electric fields induced by TMS or tDCS (e.g., Thielscher et al., 2015; Huang et al., 2018). These applications can produce an accurate model of the human head and brain in less than 3 h. However, simulations of tsDCS require a full model representing the human trunk and neck, since the tissues between the stimulus source and the spinal cord deeply impact the electric field that reaches the target. These are not simple models to obtain directly from MRI, requiring long efforts in segmentation and processing of surface representations, until a full model is adequate for numerical simulations (Christ et al., 2010; Parazzini et al., 2014; Fernandes et al., 2018).

This narrative review aims to present the current state-of-the art on how computational constructs of NISS are developed, from model generation to electric field simulations. The main contributions and challenges of NISS computational models will be discussed at the light of protocol optimization toward more targeted applications for spinal rehabilitation.

## 2 How to build a realistic spinal model

In NISS techniques, the effect at target is strongly shaped by the anatomical morphology of target and surrounding tissues, combined with characteristics that arise for each tissue specific electrical nature (Fernandes et al., 2018; Fernandes et al., 2019a). Thus, models need to be designed as close as possible to the real tissues in the human body, reproducing the morphological relation between spinal cord, vertebral column, trunk and neck (Parazzini et al., 2014; Kuck et al., 2017; Makarov et al., 2019). As for non-invasive brain stimulation modeling, the design of realistic models for NISS is based in medical imaging data, from which high-resolution magnetic resonance images (MRI) are the most used (Christ et al., 2010; Valošek and Cohen-Adad, 2024).

**Figure 1:**
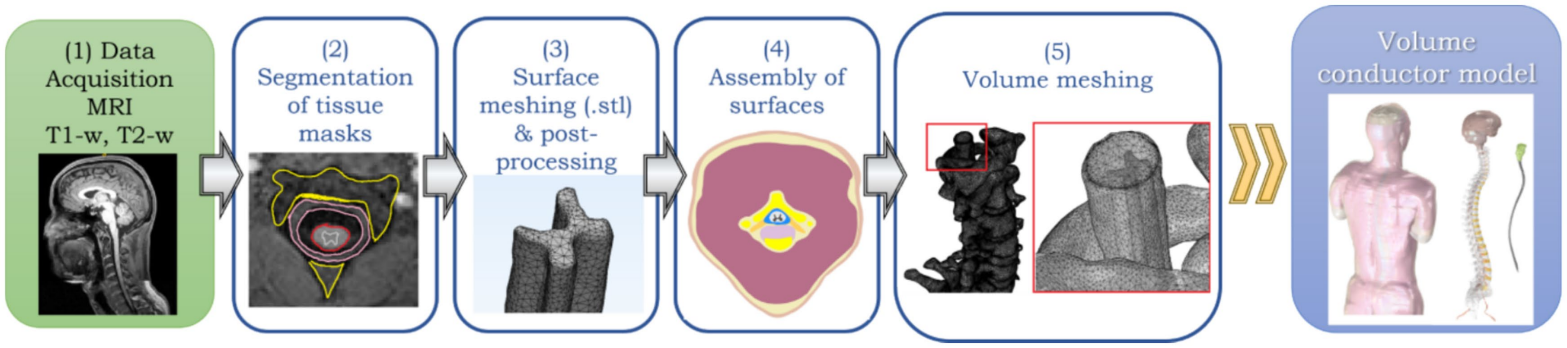
General workflow to obtain a realistic model of the human vertebral column and surrounding tissues for NISS simulations: (1) MRI data acquisition; (2) Segmentation of tissues from MRI data; (3) Surface meshing and post-processing; (4) Non-manifold assembly of surfaces; (5) Volume meshing of surface assembly to obtain a final volume conductor model ready for FEA-based simulations of NISS.

The general workflow to obtain a realistic human model for NISS is summarized in Figure 1: (1) acquire high-resolution images (e.g., MRI scans); (2) segmentation of the images to get tissue masks; (3) Convert tissue masks to surface meshes; (4) assembly surfaces into a volume; (5) generate a volume mesh to obtain a volume conductor model.

### 2.1 Image-based spinal cord models: approaches and challenges

NISS modeling inherits most of the framework built for non-invasive brain stimulation, since the final goal is to obtained an accurate representation of the spinal cord and surrounding tissues. In brain models, usually a high-resolution T1-weighted MRI (T1-w) structural scan is sufficient, using a low readout bandwidth to ensure good signal-to-noise ratio and applying a fat suppression method (e.g., selective water excitation) to minimize the signal from spongy bone, typically ensures a high quality of the resulting head models. Including a T2-weighted scan (T2-w) is recommended to help better distinguish between bone (skull) and cerebrospinal fluid (CSF), brighter in this type of scan. Accurate segmentation of CSF is essential because of its high conductivity (Saturnino et al., 2019).

#### 2.1.1 Obtaining full-body MRI scans

The same applies to spinal cord modeling, however spinal structures are much smaller. Spinal white matter (spinal-WM) can range from 6 to 12 mm of diameter, with an inner spinal gray matter (spinal-GM) even smaller. Spinal roots also have mm-order thickness (Frostell et al., 2016). To get MRI scans required for step (1) of our modeling framework (Figure 1), resolutions at least one order of magnitude smaller than in brain modeling are required to allow a detailed segmentation of spinal roots, spinal-WM and GM. The first full human models available, the Virtual Population Family from ITIS Foundation (Christ et al., 2010, itis.swiss/virtual-population/) were obtained from segmentation of MRI data taken with a 1.5 T scanner, with resolutions that varied from 0.5 × 0.5 × 1.0 mm^3^ to 0.9 × 0.9 × 2.0 mm^3^ voxel size, resulting in approximately total scanning time of 6 h, difficult to endure in the clinical or even in the research context. These first models had only some spinal roots and nerves represented and no distinction between spinal-WM and GM. Some improvements have been made to these models, having now 18 different human models, representing an age span from 8 to 84 years old, including three pregnant women at different stages. More than 300 tissues were segmented with a slight increase in resolution in the axial plane, to 0.5 × 0.5 × 0.5 mm^3^ (Gosselin et al., 2014). MRI scanners of 3 to 7 T are now available, which allow more accurate models.

The main obstacles to overcome are the costs and time required for acquisition. Obtaining a full-body high-resolution T1-w (at least 0.5 × 0.5 × 0.5 mm^3^ voxel) will require much more than 10 min acquisition, which can cause discomfort to the volunteer inside the scanner. As spinal-WM and muscle have an electrical anisotropic nature, i.e., electrical currents have preferential pathways along the alignment of fibers, diffusion tensor imaging (DTI) can improve accuracy of the model when passing on simulations of electric field effects. In fact, introducing anisotropic biophysical assumptions increases the influence of anatomy in the predicted electric field spatial patterns (Kuck et al., 2017; Fernandes et al., 2018). Including DTI would increase both the time inside the scanner and the costs involved, which may surpass 1 k euros for an individual, which challenges personalized modeling approaches, considering typical funds available for research and clinical use. One solution is to resort to datasets already acquired that can provide a good match to the population and spinal target of interest. Some examples of datasets are provided by Valošek and Cohen-Adad (2024). These datasets do not reproduce a full body image, but can provide useful information on anatomical structure of spinal-WM and GM and refine model design when a full-body acquisition at high-resolution is not possible. Combining low-resolution imaging of a full body with high-resolution acquisitions at the spinal cord can save time and resources and still provide a full body model, as already done successfully for more accurate predictions of tsDCS effects in animal models (de Oliveira Pires et al., 2025).

#### 2.1.2 Segmentation of tissues for a full body model

Obtaining tissue masks in step (2) of Figure 1 from a MRI dataset is not a simple task. In the case of brain stimulation modeling, there are some pipelines that perform automatically the workflow represented in Figure 1, when using a high-resolution MRI of the head as an input and obtaining a full head model with high anatomical detail of sulci and gyri in the cortical GM. On example is the CHARM pipeline in SIMNIBS open-source software (Puonti et al., 2020). Regarding the spinal cord, it is possible to have an automatic segmentation with clear distinction between the spinal-WM and GM from cervical to lumbar levels, including also quantitative analysis of features associated with pathologies, using the open-source spinal cord toolbox (SCT, Valošek and Cohen-Adad, 2024).^1^ However, this has to be combined with a segmentation of spinal-cord surround tissues, such as vertebrae, disks, muscles, lungs, fat and skin, since these will have an impact on current spread when applying non-invasive stimulation (Parazzini et al., 2014). There are tools available for segmentation of medical images, such as MRI or Computer Tomography (TC) which can be used for this purpose. One example is MIMICS,^2^ a commercial software licensed for clinical use, providing a GUI interface that allows visualizing the data set at 2D and 3D and performs manual and semi-automatic segmentation procedures, using techniques like thresholding or region growing. ITK-snap is an open-source analog regarding segmentation, providing tissue masks of reasonable quality.^3^ Segmentation process still require manual correction procedures in the case of the spinal cord, because semi-automatic tools strongly rely on clear resolution of features that may not be entirely visible in a full-body acquisition. This has been overcome in brain pipelines such as CHARM mentioned above, by resorting to a head template, consisting on a very accurate model in which the image is registered and used to complete the information on tissues morphology and shape not available in the source data under segmentation (Huang et al., 2018; Puonti et al., 2020). The SCT also resorts to a spinal template to obtain an accurate physical description of spinal-GM laminae and WM tracts. The design of a full-body template should be a future goal toward the automatic design of full body and spine accurate models based on each individual MRI data.

One alternative to segmentation is to use masks already available such as the ViP models mentioned above. Tissues or details that are lacking can be added artificially. For instance, in our previous works, we use the first ViP models from Christ et al. (2010), which did not distinguish between GM and WM. Based on anatomic atlases and on the Visible Human Data Set,^4^ we were able to determine dimensions and shapes of the spinal-GM relative to the spinal-WM and get an artificial design (Fernandes et al., 2018, 2019b). This can also be a solution for small features difficult to segment due to their small size, such as dorsal root ganglia (DRG), which can be possible neuromodulation targets, due to their role in many reflex pathways originated from the spinal cord (Pereira et al., 2022). More detailed models have been obtained for the cervical spinal cord, to study the effects of stimulation in the phrenic nerve, still based on anatomical artificial build-up (Wegert et al., 2024). With the advent of Machine Learning (ML) techniques, Convolutional Neuron Networks (CNN) are currently being applied for more accurate and high-resolution segmentation of small structures inside spinal nerves. Du et al. (2021, 2023) performed segmentation of confocal images of rat sciatic nerve with clear distinction between axons and myelin using the AxonDeepSeg open source CNN (Zaimi et al., 2018). The translation of this technique to segmentation of full body MRI scans should be explored in the near future, as it can provide more accurate segmentation of the spinal cord, nerves and adjacent tissues, toward faster and more detailed realistic model building for more accurate simulation of tsDCS biophysics.

### 2.2 Meshing type: tetrahedral versus hexahedral elements

Surface meshing represented in step (3) of Figure 1 is not considered in some approaches, when the main goal is to get only predictions of the EF and current density (J) obtained spatially in the spinal cord (e.g., Parazzini et al., 2014). In this case, model design jumps directly to step (5), i.e., volume meshing, considering only the voxels directly obtained from tissue masks segmented. This type of mesh is formed by cubic (hexahedral), voxel-based elements. The computational requirements regarding time and memory depend of the final purpose. Voxel-based meshes are sufficient for predicting the EF magnitude inside the spinal cord (Indahlastari and Sadleir, 2015). However, when models are used to predict the impact on specific circuits, there may be necessary to estimate the EF components at each spinal segment of interest, because neuromodulation effects depend on the orientation of neural targets relative to the induced field. In this case, tetrahedral models are more useful, since these include smoothed surfaces where normal and tangential orientations can be more accurately determined (Soldati and Laakso, 2020). Surface meshes represent these interfaces and are obtained by calculating the boundary of a tissue mask and representing it by a 3D surface divided into small triangular elements. These triangular elements reproduce more accurately convex and concave shapes that appear when changing from one tissue to another. These shapes will impact on boundary conditions imposed in EF estimates due to stimulation, because of variations in the normal component of the current density, which varies deeply in irregular surfaces, such as vertebrae and bones. Segmentation software usually provide surface meshes in specific formats, e.g. STL files. However, frequently these surfaces need to be manually repaired and remeshed, i.e., to refine it into more triangles and of higher quality. The 3-matic module from MIMICS performs automatic surface correction operations and remeshing.^5^ It also allows to edit the surfaces manually, which will frequently be the case. Gmsh^6^ and MeshLab^7^ are two open-source options that can also allow for generating and editing surface meshes, although not as user-friendly as 3-matic. Gmsh can be used with python or matlab scripting, which can be an advantage in the design of pipelines for volume model conductor generation, such as is the case of SIMNIBS (Thielscher et al., 2015).

Surface meshes need to be converted in a volume mesh in order to simulate the effects of the EF induced by NISS techniques. This conversion requires that surfaces have clear intersections or are embedded one into the other, like “russian dolls,” in order to proceed to step (4), the non-manifold assembly of surfaces (NMA). NMA “tells” the model what regions correspond to each tissue and requires a clean definition of boundaries, without intersections or overlaps. This is essential for volume meshing. Some overlaps between tissue may be allowed, but in the end these can provide errors or even result in failure during the procedures.

Volume meshing of triangle-based surfaces result in tetrahedra-based volume meshes. These are used in most simulation pipelines, such as SIMNIBS or Sim4Life. These provide more accurate estimates of EF components, which can be decisive to interpret possible effects at network and cellular level, strongly dependent on vector orientation. Tetrahedral meshes of a full-body model can take 3 to 6 h to obtain after NMA procedures, depending on mesh refinement and the number of final elements. A accurate volume conductor model of the spinal cord and trunk, can have up to 10^7^–10^8^ elements of medium to high quality, regardless of being voxel or tetrahedra-based (e.g., Fiocchi et al., 2016; Makarov et al., 2019).

What should be the type of mesh to select? Regarding EF estimations, both voxel-based and tetrahedral models suffer from artifacts in the evaluation of maximum values. In voxel-based models, numerical errors are caused by the staircase approximation errors that originate when curved boundaries are approximated with voxels (Laakso and Hirata, 2012). Even though tetrahedral meshes are not affected by staircase approximation errors, they rely on the geometrical quality of the tetrahedra. Despite having no effect on the overall EF distribution, poorly-shaped elements (irregular tetrahedra) produce artifacts that make the maximum EF unreliable. A quantitative comparison of modeling accuracy and element quality in tsDCS studies is thus essential to guide decision making regarding the type of mesh, being a major gap in the field. Even in NIBS, literature is scarce. Soldati and Laakso (2020) compared the EF predicted using four voxel grids (meshes) and four tetrahedral meshes of different resolutions. Accuracy of voxel-based meshes was evaluated by the size of the cubic element, as in tetrahedral meshes, accuracy was ascertained by three metrics of tetrahedra quality, ranging from 0—poor to 1—best quality: rho—ratio between the shortest and the longest edge of the tetrahedron; eta—ratio between the tetrahedral volume and the sum of its edge lengths; gamma—relationship between the tetrahedral volume and its total surface area. Elements of edge length of 2 mm in both mesh types cleaned most of the artifacts generated in the EF estimations. In tetrahedral meshes, this corresponded to a rho metric > 0.3. In tsDCS, element quality is usually set to a minimum of 0.3 before obtaining the volume mesh, using the knowledge acquired from NIBS models.

Regarding differences in EF estimates, these could reach 2–3% between tetrahedral models of different quality, as for voxel-based models, EF differences rise up to 10% with a 4-fold decrease of the edge length. Differences between EF estimates are around 1–2% between finer meshes regardless of the type of element (voxel or tetrahedron).

**Figure 2:**
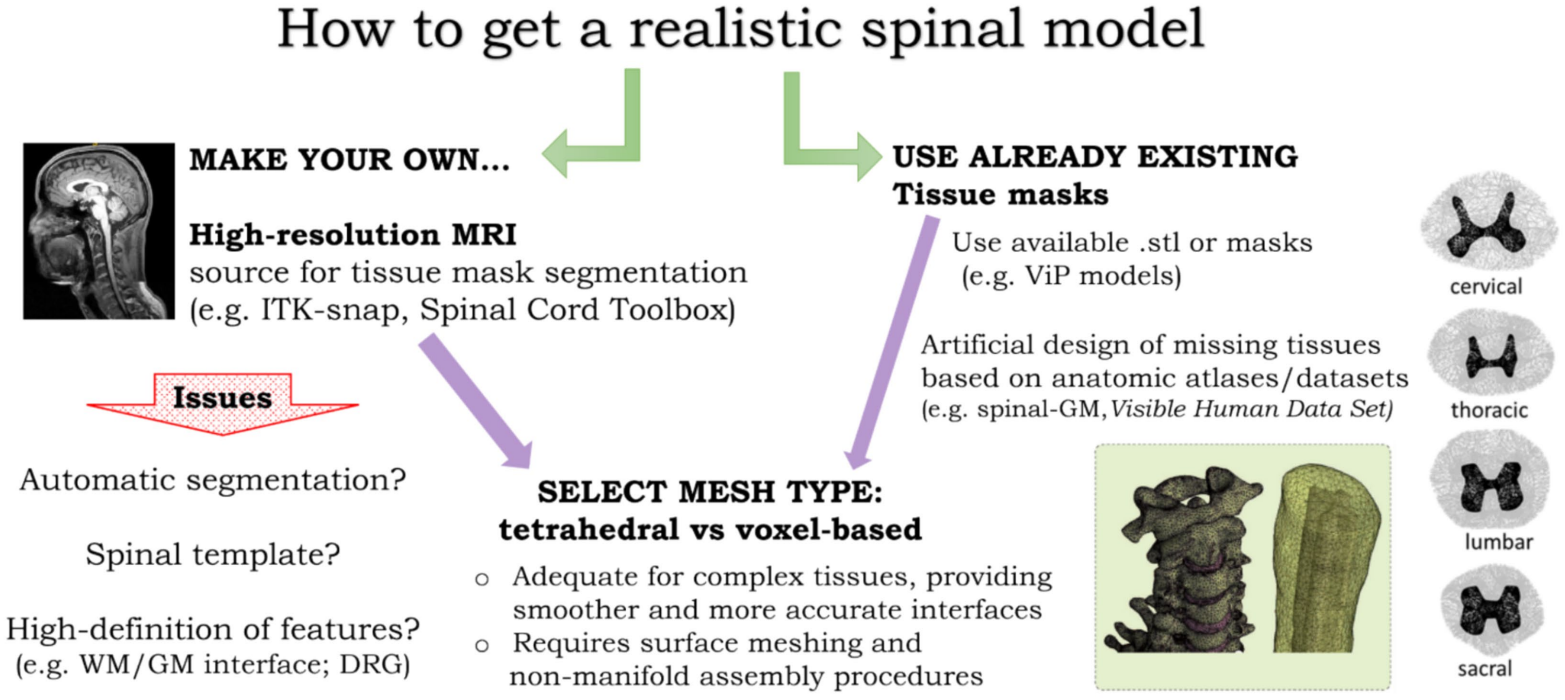
Options to consider in human model design for NISS simulations. A realistic human model requires two main options: first, obtain our own model from MRI data (left) or use already existing masks from available datasets, requiring artificial design of relevant structures, such as the gray matter tissue masks (right); second, select the mesh type, taking into account a balance between accuracy and time or computational constraints (center). Images adapted from Thielscher et al. (2015, left) and Miranda et al. (2017, right).

The decision-making on the type of approach for NISS modeling depends on the purpose and accuracy intended. Figure 2 summarizes some of the options addressed in the previous sections and issues to take into account. Regardless of choices made, there are always specific procedures to take during model design or adjustments to be made to already built-in models that depend on the specific research question to investigate with NISS computer simulations. Future studies should also be undertaken to quantify how resolution and quality of human models impact on tsDCS-induced EF distribution and orientation, as well as on computational effort. This can be decisive in model-guided clinical applications, where time and computational resources may be limited.

## 3 Simulating tsDCS in realistic models—the FEA approach

**Figure 3:**
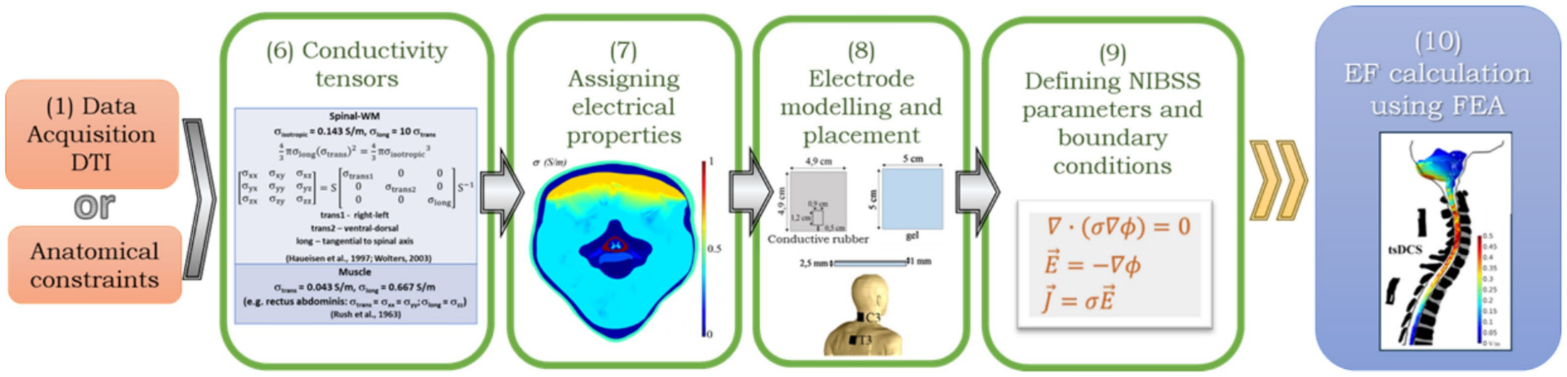
General workflow to predict the EF induced by NISS using the FEA method, proceeding from the tasks for model generation: (6) Define conductivity tensors, using information from DTI data or anatomical constraints; (7) Assign electrical properties of tissues and materials (electrodes, gel, saline solution); (8) Electrode modeling and placement; (9) Define NISS parameters, such as current or electric potential, boundary conditions and biophysical equations to calculate the EF using the FEA (*σ*, electrical conductivity; *ϕ*, electric potential; ∇, vector gradient; $\vec{E}$ , EF vector; $\vec{J}$ , current density vector.

After having a 3D human volume conductor model, the next step is to predict the EF induced in the spinal cord due to tsDCS. Input information for accurate computer simulations of NISS effects should include an accurate definition of tissue electrical properties, the stimulation parameters (e.g., electrodes’ placement, current applied) and the equations that described the biophysics of the stimulus applied (e.g., Laplace’s law, current conservation). The workflow involved in setting and running a simulation in tsDCS is represented in Figure 3. The main tasks involved are, following from Figure 1: (6) characterize isotropic/anisotropic nature of tissues regarding conductivity; (7) assign electrical properties to tissues in the model and electrode’s materials; (8) model the geometry and placement of electrodes; (9) Setting up the biophysical equations, with initial and boundary conditions; (10) Perform the FEA to determine the EF at each element in the model.

### 3.1 Electrical behavior of tissues and materials

Electrical properties of tissues can impact deeply the properties of tissues. First works in tsDCS considered tissues to conduct isotropically, i.e., conductivity has the same behavior regardless the direction of the current. However, tissues that are composed by fibers oriented in a particular direction - muscle, spinal tracts and spinal-GM laminae – have an anisotropic nature. For these tissues, electrical conductivity (*σ*) should be defined in terms of a tensor, to adequately express changes of σ according to direction. Diffusion Tensor Imaging (DTI) can be a powerful tool to determine accurately σ tensors for electrical excitable tissues in the human body, by indicating the correct alignment of fibers and assigning the value of conductivity merely in the specific orientation at each mesh node (Wenger et al., 2015).

When DTI is not available, some theoretical assumptions can be made, for example, by considering that spinal-WM has higher conductivity along the caudal-rostral direction due to the orientation of spinal tracts being almost coincident with the spinal canal axis. Wolters (2003) proposes a volume constraint for neural fibers, which relates longitudinal and transversal conductivities by Equations 1, 2:


$$\sigma_{\text{long}}=10\;\sigma_{\text{trans}}$$ (1)

$$\frac{4}{3}\pi \sigma_{\text{long}}{\left(\sigma_{\text{trans}}\right)}^{2}=\frac{4}{3}\pi {\sigma_{\text{isotropic}}}^{3}$$ (2)

To correctly assign anisotropic conductivity to each element in the volume conductor model, a diagonal matrix can be defined considering a local coordinate system of one longitudinal conductivity (caudal-rostral, $\sigma_{\text{long}}$ ) and two orthogonal transverse conductivities ( $\sigma_{\text{trans}}$ ) of equal value (e.g., medial-lateral and ventral-dorsal). This matrix can be rotated to the model’s reference coordinate system (x, y, z) using a transformation matrix S for each mesh node. A similar approach can be done for muscle tissue, taking into account the orientation of each muscle according to the direction of muscles fibers known from anatomy.

The differences in EF predictions considering anisotropy in the model lie essentially in local maxima caused by anatomical features, such as spinal canal narrowing due to bone or intervertebral disks protrusions, which are not so evident using only isotropic conductivities for the spinal-WM (Fernandes et al., 2018). The same observations were made in brain stimulation models. Anisotropy modeling can highlight the influence of anatomical differences, so it may be relevant to understand interindividual variability usually observed in stimulation effects.

For isotropic tissues, a scalar conductivity can be assigned to each tissue domain. First modeling studies in tsDCS (e.g., Parazzini et al., 2014; Kuck et al., 2017) were based on the list of dielectric properties of tissues determined by Gabriel et al. (1996, 2009), provided for the ViP models. Gabriel et al. (2009) refers that, at frequencies below 100 Hz, these conductivity values may be underestimated due to the assumptions made to correct for electrode polarization effects. For example, *σ*(spinal-WM) = 0.027 S/m while σ(bone) = 0.020 S/m, which is not realistic since bone is not a conductive tissue, thus its conductivity should be much lower that WM. Different electrical conductivity assumptions can lead to different estimates and have a strong impact in magnitude and spatial patterns near currents’ region of entry – i.e. over electrodes placed over the spinal cord (Fernandes et al., 2018).

### 3.2 Modeling tsDCS delivery—electrodes

Within the modeling framework, the next step is to introduce the media that induces the stimulation, i.e., the electrodes. tsDCS electrodes are the same used in cortical stimulation, made of conductive rubber, with a metallic connector to transmit current from a source and embedded in a saline-soaked sponge. Alternatively, a layer of conductive gel can be used to provide a safe contact surface between the electrode and the skin (Fernandes et al., 2019b). The shapes can vary from rectangular to squares or disks.^8^^,^ ^9^

Most computational studies model electrodes as a simple rectangle of saline-soaked sponge or gel (Parazzini et al., 2014; Bastos et al., 2016; Fiocchi et al., 2016; Kuck et al., 2017). However, NIBS simulations show that small differences in the cortical EF distribution are predicted under the electrodes connectors and edges when comparing models with and without connectors (Saturnino et al., 2015). In our first study (Fernandes et al., 2018), we modeled tsDCS electrodes with connectors and varied connector relative position between anode and cathode. Electrode placements with connectors placed distally to each other produced slight changes in the spinal EF magnitude, 3–7% higher in segments under or near connectors. This effect is weaker for placements with only one electrode on the spinal cord. As typical values of EF are less than 1 V/m, these changes are less than 0.01 V/m, and thus may not be sufficient to introduce noticeable variations in real neuromodulation effects. Nevertheless, Kuck et al. (2017) predicted changes in neuron polarization due to lumbar and thoracic tsDCS when simulating displacements of 5 cm on electrode positions (Kuck et al., 2017). The shape of the electrodes is especially relevant, since rectangular or square electrodes result in local current hotspots in the corners, an effect that is not seen when using circular electrodes.

These questions are relevant because assembly of electrodes into the model usually have to done before NMA during model generation (Figure 1). The intersection of the electrode domain with the skin may generate overlaps that cause failure of the volume mesh procedure. A simplified model of electrode may facilitate this process. So, to get a reasonable accurate modeling of current delivery, electrodes should at least take into account the shape and the position of the electrodes, even if choosing to use a model with one the conductive layer of gel/saline-soaked sponge. One alternative is to use virtual models of electrodes, i.e., have a surface description of the effect at the interface between electrode and skin. This could be done by simply modeling the current distribution in the gel/saline—skin interface and then introduce this condition into the model at the protocol setting stage. However, as skin surface varies deeply in different regions of the vertebral column due to spinous processes and back muscle volume, this may not account for uneven distribution of current from conductive layer to skin.

### 3.3 The physics behind NISS models

Stimulation protocol setting is the final step before running a tsDCS simulation. The procedure is analogous to brain stimulation, i.e., tDCS. Biophysical calculations in the human volume conductor model are performed using the Finite Element Analysis method (FEA). The FEA is a numerical method applied in realistic modeling problems involving physics and geometry that require solving boundary value problems for partial differential equations. It considers each domain in the full model as divided into smaller domains called finite elements (e.g., tetrahedral elements). Equations governing the physics in each finite element are assembled into a larger system of equations to model the complete domain using variational methods (Reddy, 2006). FEA is a useful tool to use in bioelectromagnetic calculations since this allows a more detailed description of the volume conductor model to study considering heterogeneity and anisotropic properties of the biologic tissues included in the model (Haueisen, 1996).

The current density and EF induced in all domains of the model are calculated within the framework of Maxwell’s equations of electromagnetism. Since DC currents is constant current, it has null (or negligible) frequency, thus the quasi-statics approximation for low-frequency currents can be considered, i.e., and all tissues can be considered as purely resistive with no capacitive behavior, assuming only different electrical conductivities and setting relative permittivity (ε_r_) to 1. The main equations to solve in each node of the mesh elements are (Miranda et al., 2013; Huang et al., 2018):

- Laplace equation for the electric potential in each element ( ϕ ), $\nabla^{2}\phi =0$ ;
- gradient of the electric potential ϕ , to determine the EF vector $\vec{E}=-\nabla \phi$ ;
- Ohm’s law, $\vec{J}=\sigma \vec{E}$ , to estimate the current density ( $\vec{J}$ ), where *σ* is the electrical conductivity defined in each tissues’ nodes.

with the following boundary conditions (Miranda et al., 2013):

- continuity of the normal component of the current density in all interior boundaries (current conservation);
- electric insulation in the external boundaries;
- electrode connectors were considered as isopotential surfaces.

DC sources are usually current-controlled, so potential difference between the anode and cathode is adjusted in simulation pipelines considering a floating potential boundary condition at the surface where the current is injected—top surface of layer between electrode and skin or connector surface if represented in the model (Salvador et al., 2010; Miranda et al., 2013).

Analysis of results will depend on the purpose. First, how much EF is required at the spinal target of interest to ensure neuromodulation? There is no define EF threshold for neuromodulation, since invasive measurements are not ethically feasible. However, some assumptions can be made when matched neuromodulatory effects observed with EF magnitude predicted. Simulations in tDCS predicted an average EF magnitude in the motor cortex at the hand knob higher than 0.15 V/m, when applying 1 mA to the scalp and reproducing the same conditions mentioned in tDCS clinical trials (Miranda et al., 2013). Simulations of the EF in tsDCS using the same rationale result in EF magnitudes > 0.20 V/m with neuromodulatory effects observed (Kuck et al., 2018; Fernandes et al., 2019b). However, neuromodulation does not depend only on EF magnitude but also on alignment of the vector field relative to the neural target and its specific properties, since activation thresholds vary among neuron types (Reato et al., 2019). This leads us to the second consideration on analysis. To predict neuromodulation in spinal neurons or networks it may be necessary to consider EF components. Since alignment of neural targets varies with the orientation of the spinal axis, a local coordinate system is recommended, considering a caudal-rostral axis, tangent to the spinal axis at every segment of interest, and two orthogonal axes, one ventral-dorsal, the other medial-lateral. This may require matrix transformations at each spinal node, which are usually done with custom-made programming scripts in matlab or python (Fernandes et al., 2018; Kuck et al., 2017; Thielscher et al., 2015).

Although the FEA has been the method applied to estimate the tsDCS-induced EF in realistic models, other methods can be considered. For example, Makarov et al. (2019) applied the boundary element fast multipole method (BEM-FMM) to predict the EF induced by Transcutaneous Electric Nerve Stimulation (TENS) in a full tetrahedra-element based full body model. BEM operates directly with the surface meshes, it is based on electric charge at boundaries (instead of electric potential) and does not require an artificial boundary or volumetric tessellation nor NMA operations, thus making computational time and resources lighter (Makarov et al., 2018). Du et al. (2021, 2025) proposes the Admittance Method (AM), using python-based open-source development (AM NEURON), to estimate current density and EF due to Peripheral Nerve Stimulation (PNS). This method is applied at each node of voxel-based nerve models to determine the voltage from an estimated admittance (G) matrix. Given the conductivity values of tissues composing the model, it is possible to estimate the voltage at each node and its neighbors, regardless of differences in voxel dimensions from tissue to tissue. This method allows to perform simulations at different resolutions, with a strong potential in tsDCS simulations using full body models, where tissues vary in resolution from very detailed nerve and spinal cord structures to large tissues, such as muscles and bones.

### 3.4 Software applied for tsDCS modeling

There is no software pipeline that provides full segmentation of MRI data, volume conductor model generation and tsDCS simulations using FEA. There are some tools available online that can be combined to obtain a model and then simulate the effects of tsDCS. Some of the most frequently used tools are presented in Table 1. In our previous approach, models were built with 3-matic model from MIMICS, taking approximately 6 h to get a viable volume mesh conductor model. This model was imported to COMSOL Multiphysics software (Table 1), in which a tsDCS simulation was configured using the AC/DC module. When run, the simulation resulted in 2.6×10^7^ degrees of freedom and the solution time was about 150 min, using a computer with 2 quad-core Intel® Xeon® processors clocked at 3.2 GHz and 48 GB of RAM (Fernandes et al., 2018; Fernandes et al., 2019a). Most clinical settings do not have computers with these characteristics, so faster and lighter solutions should be considered in the future (for example, BEM-FMM, AM NEURON, see section 3.3).

**Table 1:** Software used for full body model design and/or tDCS simulation, with indication of the type of procedures available (✓) or not (×) according to the workflow indicated in Figures 2, 3: Segmentation of tissues; surface and volume meshing; EF simulation; availability (open, open source, or commercial—license required); issues/limitations identified for the operations referred in each software.

Name	Segmentation	Meshing	EF simulation	Availability	Issues
Spinal Cord Toolbox (1)	✓	×	×	Open	Segmentation of spinal cord GM and WM (only)
ITK-snap (2)	✓	×	×	Open	Manual procedures required
Gmsh (3)	×	✓	×	Open	Limited capability
ScanIP (4)	✓	✓	×	Commercial	Manual procedures required
MIMICS (3-matic) (5)	✓	✓	×	Commercial	Manual procedures required
COMSOL (6)	×	✓	✓	Commercial	Only performs successful meshes of geometric representations
Abaqus (7)	×	✓	✓	Commercial	Only performs with geometric meshes
SEMCAD X (8)	×	✓	✓	Commercial	Uses tissue masks of available models
Sim4Life (9)	×	✓	✓	Commercial	Uses tissue masks of available models

(1) https://spinalcordtoolbox.com/; (2) http://www.itksnap.org; (3) https://gmsh.info; (4) https://www.synopsys.com/simpleware/software/scanip.html; (5) https://www.materialise.com/en/healthcare/mimics-innovation-suite; (6) https://www.comsol.com; (7) https://www.3ds.com/products-services/simulia/products/abaqus/; (8) https://speag.swiss/products/semcad; (9) https://zmt.swiss/sim4life.

Open source tools—e.g. ITK-snap, spinal cord toolbox—can eventually be combined with NIBS simulations such as SimNIBS or ROAST to have a full pipeline for model design and simulation, with some complex programming scripting, which is still under construction and poses many challenges, such as file format conversion. For example, it is possible to use SimNIBS software to run models different from head models, but meshes should be converted to a compatible format (Camera et al., 2024, Professor Axel Thielsher, private communication). This process may take hours, especially with large meshes (10^7^–10^8^ elements). Commercial solutions can be more user-friendly, such as is the case of Sim4Life. Although not integrating full segmentation, Sim4Life has the ViP model dataset, which reproduce different human phenotypes, allowing for more realistic simulations, by selecting the model closer to the subject’s anatomy of interest. Simulation settings are more accessible, requiring only basic knowledge at computer user’s level. This is the most complete simulation pipeline available for tsDCS at the moment, however it will depend on availability of funds to obtain a user’s license (Gosselin et al., 2014).

## 4 What do models tell us? Impact of NISS models in science and health

Studies on tsDCS effects dedicated first to simulate experimental studies using electrode montages with one electrode over the thoracic spinal processes T10, T11 (e.g., Cogiamanian et al., 2008; Hubli et al., 2013), to find possible neural correlates with EF distribution. Soon after, simulation studies inspired new electrode montages, with both electrodes placed over the spinal cord, in an attempt to focus stimulation in the spinal canal and increase effects. One good example was the methodology adopted by Kuck et al. (2018), in an experimental study with tsDCS fully delivered over the vertebral column, supported by the findings of previous modeling work (Kuck et al., 2017). This methodology can have two main contributions: (1) optimize EF delivery at a spinal target by rehearsing protocols in a virtual bench (computational model); (2) find neural correlates of tsDCS-induced EF from neurophysiological readouts from experimental studies guided by tsDCs modeling.

One example of contribution (1) is from Kuck et al. (2018): an experimental study was carried comparing the effects of one-vertebral electrode (S) over T11 spinous process (sp) versus two-vertebral electrodes equally disted (ED) from T11sp. Only ED montage resulted in amplitude reduction of the H-reflex with respect to baseline. This goes in line with modeling work demonstrating a higher EF when using both electrodes over the vertebral column. Contribution (2) can be inferred from the original work published by our group comparing four electrode montages over the spinal cord for modulation of cervical motor-related responses (Fernandes et al., 2019b). Taking a deeper look into the results, we can observe than the weight of components in total EF magnitude, presented in Table 2, does seem to impact on the type of neuromodulation changes observed in the corresponding experimental studies. Cervical montages with a higher contribution from the longitudinal, caudal-rostral EF component (E_long_, caudal-rostral) resulted in neuromodulation of time-dependent responses (e.g., peripheral silent period—PeSP; central motor conduction time—CMCT). Changes in amplitude of motor-evoked responses (MEP) occur in montages with a ventral-dorsal EF component (E_vd_) comparable or higher than E_long_. These findings show that neuromodulation depends on relative orientation between the EF and neural targets as predicted by theory (Reato et al., 2019).

**Table 2:** EF components and neuromodulation effects in four experimental studies in cervical tsDCS.

Montage	Stimulation protocol	Outcomes	EF_component_/EF_magnitude_			Reference
			E_long_	E_vd_	E_rl_	
C7 rD	I = 2.5 mA Δt = 20 min J = 0.071 mA/cm^2^ Q = 85.7 mC/cm^2^	Increased MUNE and decreased PeSP with cathodal polarity	**0.91**	0.01	0.05	Bocci et al. (2014)
C7 CMA	I = 2.0 mA Δt = 20 min J = 0.071 mA/cm^2^ Q = 85.7 mC/cm^2^	Increased MEP amplitudes independent of polarity	**0.66**	**0.59**	0.08	Lim and Shin (2011)
	I = 3 mA Δt = 10 min J = 0.1 mA/cm^2^ Q = 60 mC/cm^2^	Acute effects on electrical elicited C-MEP: amplitude increased in cathodal and decreased in anodal				Dongés et al. (2017)
C3-C5 CMA	I = 2.5 mA Δt = 15 min J = 0.071 mA/cm^2^ Q = 63.9 mC/cm^2^	Increased Di-MEP amplitudes independent of polarity; Increased tidal volume with cathodal stimulation	0.26	**0.86**	0.02	Niérat et al. (2014)
C3 T3	I = 2.5 mA Δt = 15 min J = 0.071 mA/cm^2^ Q = 63.9 mC/cm^2^	Decrease in MEP latency and CMCT with cathodal polarity	**0.87**	0.15	0.12	Fernandes et al. (2019b)

Montage—positions of electrodes (anode, cathode); Stimulation protocol—current settings and time duration; Outcomes—effects observed in upper limb neurophysiological responses; EF_component_/ EF_magnitude_ - ratio between EF components’ Elong, Evd, Erl (from left to right) and total EF magnitude; Reference of the study. Positions over the spinal cord are over spinous processes of corresponding vertebra. Outcome cells are in blue and green to highlight time and amplitude related modulation effects. C, cervical; Di, Diaphragmatic; T, thoracic; I, current intensity; Δt, duration; EF, electric field; J, current density; Q, total charge; CMCT, Central Motor Conduction Time; MEP, Motor-Evoked Potential; MUNE, Motor Unit Number Estimation; PeSP, Peripheral Silent Period; long, longitudinal; vd, ventral-dorsal; rl, right–left; rD, right deltoid muscle.

### 4.1 Fine-target optimization: high-definition tsCS

**Figure 4:**
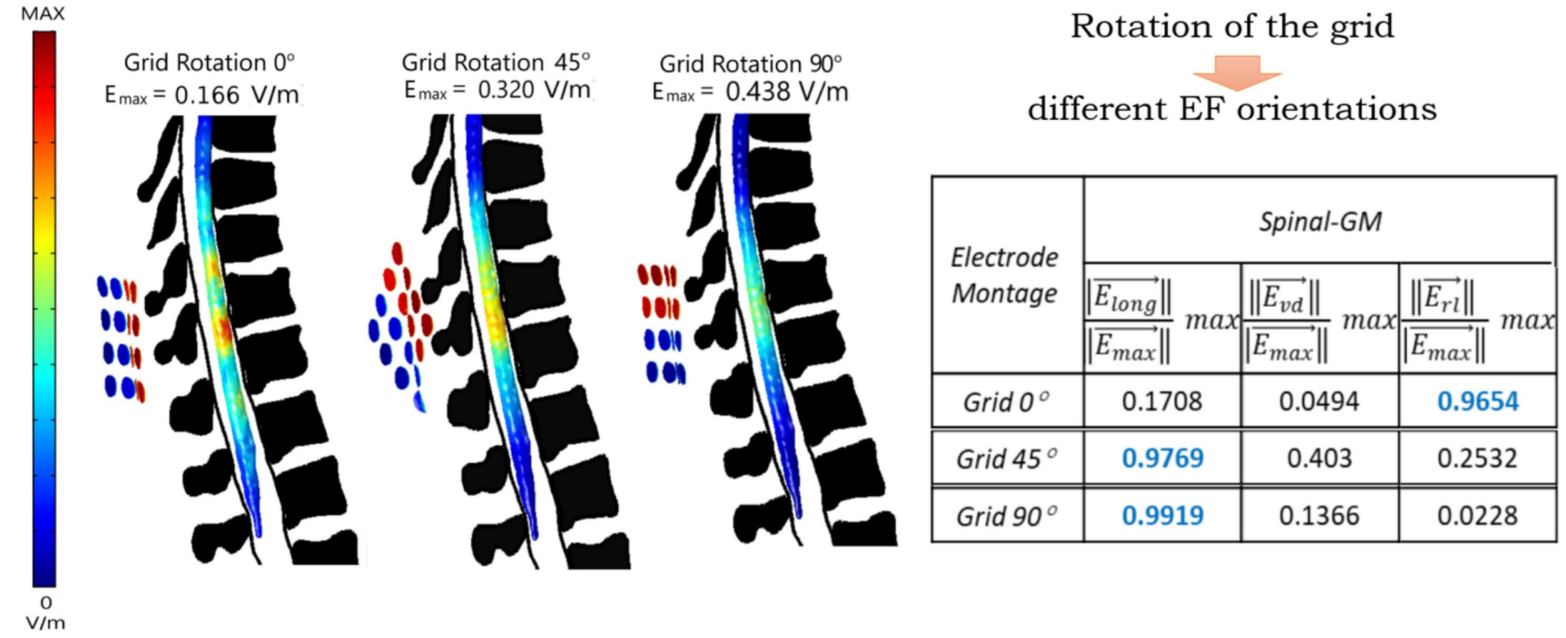
Computer simulations considering a 4-by-4 multielectrode grid results in different contributions of the EF components when rotating the grid [adapted from Fernandes et al. (2023)]. Left: EF magnitude distribution predicted in the thoraco-lumbosacral spinal cord for a grid placement between T9–T11 s.p. and grid rotations of 0°, 45°, and 90°. Anodes and cathodes are represented by red and blue circles, respectively. The color scale represents the EF magnitude normalized to maximum. Right: maximum value of the magnitude of EF components relative to maximum (max) total field for each grid rotation, with larger values marked in blue (long, longitudinal; vd, ventral-dorsal; rl, right–left). Different orientations can result in different modulation effects, as observed by Kim et al. (2025).

In cortical stimulation, placement of 4 or more electrodes into specific locations can lead to more localized EF in an intended target (Kuo et al., 2013; Pixa et al., 2017), being commonly referred to as High-Definition (HD) stimulation. Previous findings in tsDCS indicate that pure vertebral electrode placements contribute to more focal tsDCS delivery. Thus, could HD montages have the same type of effect in tsDCS? Grid-based systems are being considered to evaluate delivery of more targeted protocols in tsDCS. One 4-by-4 electrode system was conceptually modeled and is presented in Figure 4 [adapted from Fernandes et al. (2023)]. The same grid was applied with transverse (TRANS) and diagonal (DIAG) settings of the 4-by-4 grid system centered at the midpoint between T10 and T11 sp. (Kim et al., 2025). The soleus H-reflex and the tibialis anterior flexion reflex were measured before and after 20 min of spinal stimulation. The stimulation applied was delivered at 100 Hz. Although not tsDCS, the quasi-static approximation holds at frequencies below 100 kHz, so the same modeling framework used in tsDCS can be applied. No modulation was observed for the H-reflex for grid settings. There was an inhibitory effect on flexion reflex, regardless of grid position, but only the DIAG setting significantly shortened the duration of this reflex. HD montages can contribute for selective neuromodulation of spinal reflex networks and impact different facets of activation.

Huang et al. (2018) proposes an optimization pipeline for HD-tsDCS. An electrode system positioning was considered using a 3/9/7 system, defining the number of electrodes equally spaced in rows in the cervical, upper thoracic and thoracolumbar region, respectively. Electrodes were modeled with a circular shape and a diameter of 20 mm. The optimization pipeline was developed in Abaqus,^10^ and criteria for optimization was to calculate the montage that provides the maximum EF magnitude at target with larger focality, i.e., minor EF spread at non-target areas. Results were compared to an ad-hoc montage 4×1 ring shaped targeting T5 sp., analogous to HD montages applied in the brain. Montages optimized with the pipeline resulted in an higher EF at the intended target when compared to the ring ad-hoc montage. This work highlights the importance of automatic pipelines to provide more effective tsDCS protocols that may not be considered in ad-hoc positioning guesses done in most tsDCS modeling studies.

## 5 Toward a stimulation-wise spinal rehabilitation

Non-invasive spinal stimulation (NISS) holds significant promise as a co-adjuvant therapy for neural dysfunctions, by accelerating recovery in spinal conditions or delaying functional decline in degenerative diseases when integrated with rehabilitation strategies. Computational studies play a pivotal role in this process, guiding the optimization of electric field (EF) magnitude and orientation at specific targets, according to each patient’s phenotype. The future of NISS relies strongly in bridging the gap between modeling and experimental approaches, toward a mechanistic understanding of biophysical effects that will allow personalized targeting in clinical practice. As measurement of EFs in humans is not ethically feasible in non-invasive neuromodulation techniques such as tsDCS, validation of models seem difficult to achieve. Studies in animal models can be helpful to identify possible sources of discrepancies between modeling predictions and real EFs induced at the spinal cord during tsDCS. de Oliveira Pires et al. (2025) presented voltage gradients (EF) measurements in spinal cords of anesthetised SOD1 mice during application of tsDCS. These values were compared to EFs estimated in a computational model based on high-resolution MRI of a mouse of a similar type (C57/B6). While the EF spatial variation was similar to model predictions, the experimental value was 2 to 4 fold smaller. Electrical conductivity assumptions, uncertainty of the experimental measures and contributions of interindividual anatomy of the mice may account for this difference. Even if not directly translating to the human case, it is expected that human models can also present systematic discrepancies regarding the estimation of EFs induced by tsDCS at the spinal cord. With the development of more precise and novel MRI techniques, *in vivo* measurements of electrical properties of human tissues may be feasible in the near future, helping in more accurate predictions using tsDCS models.

Individualized models are essential to account for anatomical variability and disease-specific changes. The use of precise models of nerves revealed that electric stimulation can penetrate deeper into the tissue according to the degree of damage and/or demyelination, pressing the need for high-definition models that allow for personalization of safety criteria in protocol design, according to the specific characteristics of the disease or spinal condition (Du et al., 2025; Du et al., 2023). However, as high-fidelity models offer superior precision in predictions, these include time and computational resources not reachable or applicable in the current clinical contexts, where decision demands require often solutions at short notice. The design of a spinal template and a complete pipeline for tsDCS including segmentation, meshing, and finite element analysis (FEA), is a “MUST HAVE” for model-guided clinical approaches in spinal dysfunctions with tsDCS. Introduction of multi-scale models and combination of computational simulation strategies are essential to determine and optimize the effects of stimulation from large tissues to fiber and cellular spinal components. Additionally, network-level models that incorporate anatomy and disease phenotype can be further useful for functionally targeted stimulation. With the progression and refinement of spinal modeling pipelines, carefully validated by experimental studies with neurophysiological assessment, application of tsDCS can transition from virtual research benches to clinically robust, patient-centered approaches toward personalized rehabilitation of spinal functions.

## Footnotes

1
www.spinalcordtoolbox.com

2
www.materialise.com/en/healthcare/mimics

3
https://www.itksnap.org/pmwiki/pmwiki.php

4
www.nlm.nih.gov/research/visible/visible_human.html

5
www.materialise.com/en/healthcare/mimics/3-matic-medical

6
www.gmsh.info

7
www.meshlab.net/

8 https://www.neuroelectrics.com/; https://soterixmedical.com/

9
https://www.neurocaregroup.com/neuroconn

10
www.3ds.com/products/simulia/abaqus
